# Primary Angiosarcoma of the Central Nervous System: Case Report and Review of the Imaging Features

**DOI:** 10.5334/jbr-btr.1087

**Published:** 2016-10-10

**Authors:** Naim Jerjir, Julie Lambert, Lieve Vanwalleghem, Jan Casselman

**Affiliations:** 1 AZ Sint-Jan Brugge-Oostende AV, BE

**Keywords:** Angiosarcoma, Primary Angiosarcoma, Primary Central Nervous System (CNS) tumors, Sarcoma

## Abstract

Primary angiosarcoma of the central nervous system is a rare malignant tumor with only 28 reported cases so far. Imaging findings have only been reported in a few cases. We report a case of intracranial angiosarcoma in a Caucasian male and present a review of the imaging features in the recent literature. The tumor mostly presents as a well-demarcated, heterogeneous, moderately to strongly enhancing lesion with signs of intratumoral bleeding and surrounding vasogenic edema. The differential imaging features of common hemorrhagic intracranial tumors are discussed.

## Case Report

A 61-year-old Caucasian male with no significant medical history was admitted to the emergency department in 2006 with subacute, aggravating headaches. He had been vomiting for two days. Further clinical investigation and anamnesis revealed a mild fever of 38.0°C as well as alternating but progressive confusion and anomic aphasia for several months.

**Figure 1:**
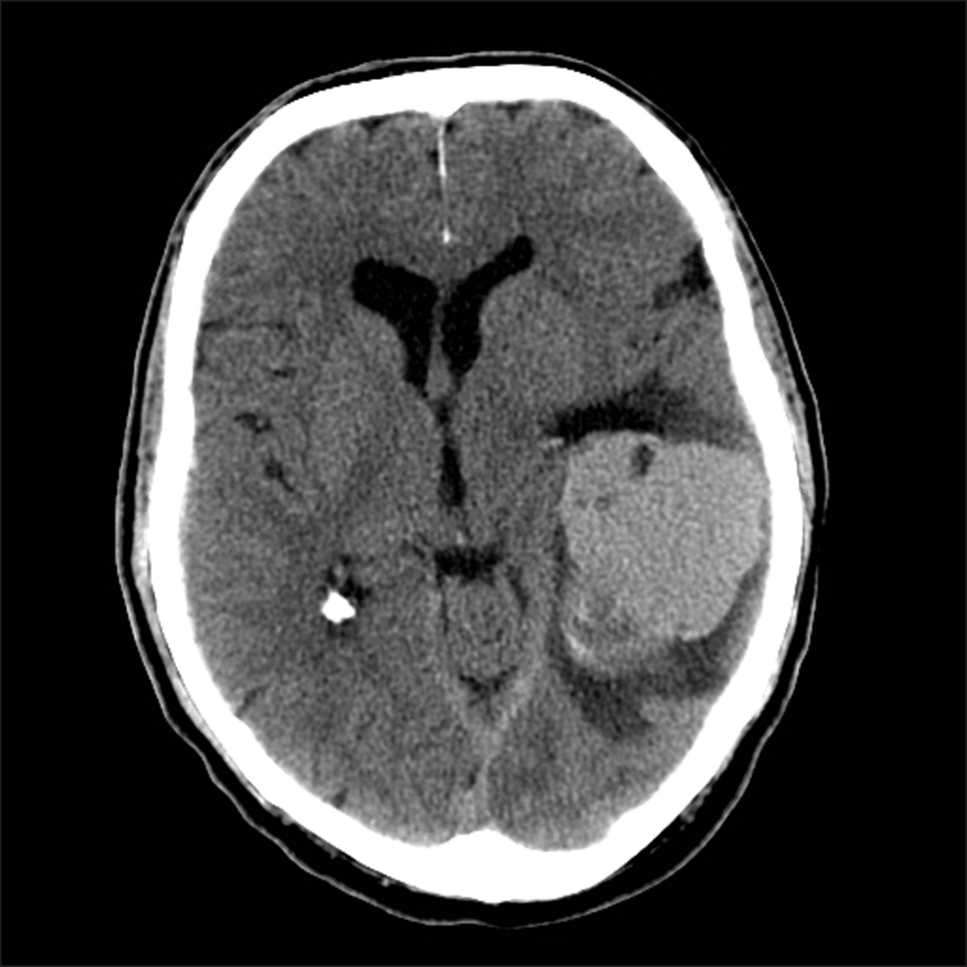
Unenhanced CT shows a well-defined, spontaneously hyperattenuating lesion in the left frontotemporal lobes. Posteriorly, a heterogeneously hyperattenuating/hypoattenuating area can be seen. Cystic/necrotic components are depicted within the lesion and vasogenic edema is surrounding the lesion.

An unenhanced CT yielded a 5.8 cm large and well-defined spontaneously hyperattenuating lesion in the left frontotemporal lobes, abutting the tentorium cerebelli, with a small component of the tumor expanding into the left cerebellopontine angle of the posterior fossa (Figure 1). Within this lesion, cystic components were apparent. The posterior part of the tumor was more heterogeneous with both hypoattenuating and hyperattenuating areas. There was mild to moderate vasogenic edema surrounding the lesion with significant mass effect, contralateral displacement of the midline, obliteration of the sulci, and compression of the left lateral ventricle with secondary dilatation of the temporal horn.

**Figure 2:**
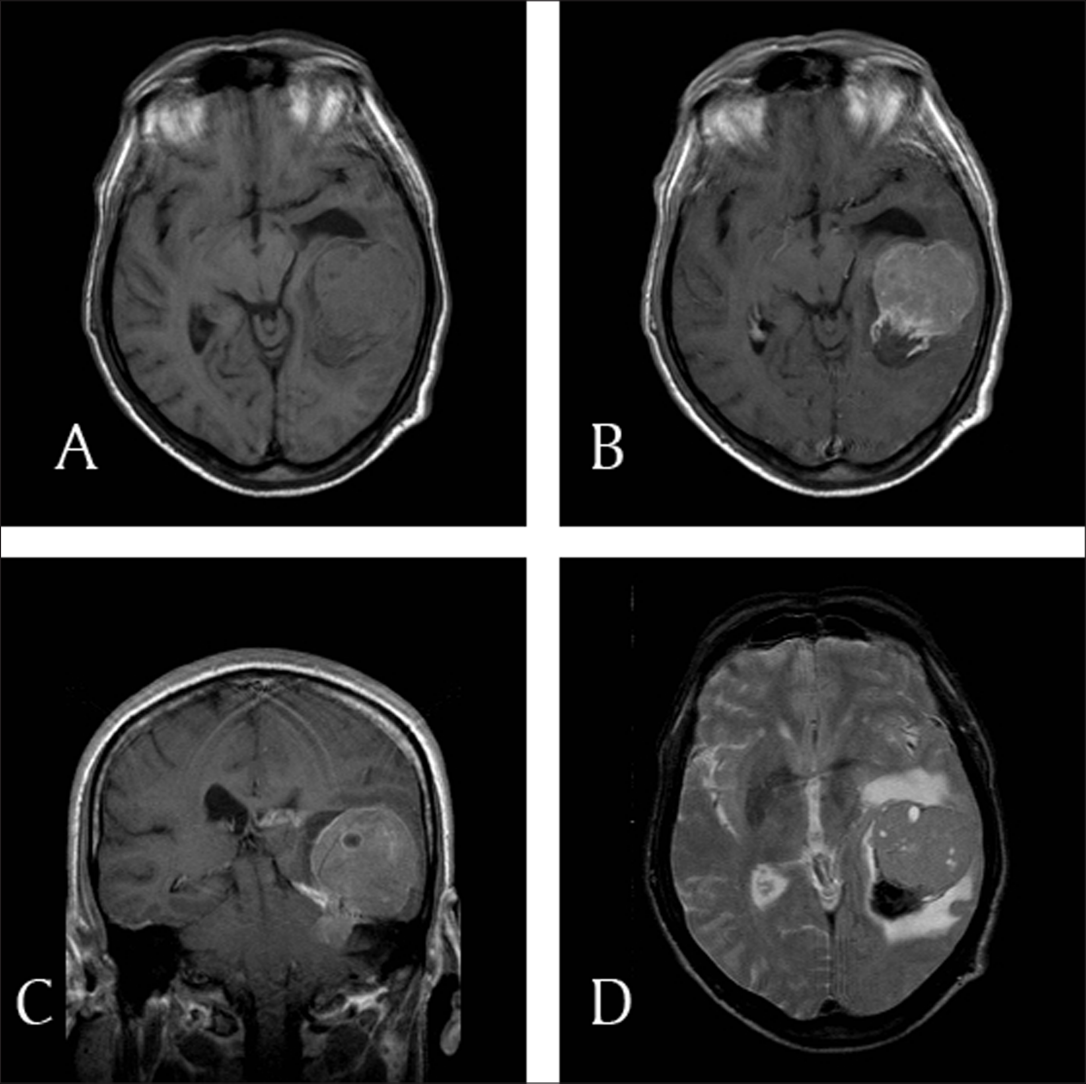
**(A)** Axial unenhanced T1-weighted image: The lesion appears isointense to grey matter image. There are no spontaneously hyperintense areas within the lesion. Dilatation of the temporal horn of the left lateral ventricle is evident. **(B)** Gadolinium enhanced T1-weighted image: Moderate, heterogeneous contrast enhancement which is more pronounced at the periphery. There is no enhancement posteriorly in the lesion. **(C)** Coronal gadolinium enhanced T1-weighted image: Rim enhancement surrounding the cystic areas can also be seen on this coronal image. A small component of the tumor extends below the thickened tenorium cerebelli in the left cerebellopontine angle. The tumor is involving the supra- and infratentorial surface of the tentorium and therefore seems to be dural based. **(D)** Axial T2-weighted image: This image is slightly more cranial than images (A) and (B) and reveals a lesion that is isointense with grey matter, with multiple cystic/necrotic areas and surrounding vasogenic edema. The posterior part of the tumor contains hypointense blood degradation products.

MRI showed that the lesion was mainly isointense with grey matter on both T1- and T2-weighted images. Multiple small T2-hyperintense and T1-hypointense cystic components could be seen within the lesion (Figure 2). No hyperintense spots could be depicted on the unenhanced T1-weighted images. Enhancement was diffuse and mildly heterogeneous, with accentuated rim enhancement both at the periphery of the lesion and surrounding the cystic areas. The posterior part of the lesion appeared strongly hypointense on the T2-weighted images and slightly hypointense on the T1-weighted images, without enhancement after intravenous administration of gadolinium. These findings most probably reflected the presence of acute bleeding. Thickening and abnormal enhancement of the tentorium cerebelli could be seen on the left side.

The treatment for this patient consisted of surgical excision with postoperative radiotherapy. The patient was still free of macroscopic recurrence on follow-up MRI 105 months after the initial diagnosis.

## Discussion

Primary angiosarcoma of the central nervous system is a very rare malignant tumor arising from the vascular endothelium of the brain and its coverings. To our knowledge, only 28 other cases have been published so far, three of which were congenital [1,2,3] and 25 developed later in life [1,4,5,6,7,8,9,10,11,12,13,14,15,16,17,18]. The average age of patients with a non-congenital tumor was 36 years, with only two patients of 60 years or older at the time of the diagnosis. Two cases in one report [19] have not been taken into account because of the extradural location of the lesions. Only one tumor originated in the spinal cord [6], the remaining 27 cases were located intracranially.

Two secondary brain angiosarcomas have been described in the literature as well, one induced by Thorotrast (a former contrast agent) and one by radiation therapy [20,21]. Both lesions appeared in patients who were older than the average age of the patients with primary angiosarcomas (68 and 67 years old, respectively). These two lesions have been included in the discussion below.

Patients most frequently present with acute clinical deterioration, sometimes coinciding with a history of vague neurological symptoms. The acute worsening of their clinical state might be due to bleeding within the tumor, as in this case. The prognosis is comprised of either one of two contrasting outcomes: death within months after surgery [1,4,5,6,7,11,13,14,15,17,20,21] or disease-free survival up until the last follow-up [1,2,3,9,12,16,18]. The patient of this report has the longest reported disease-free survival after therapy of 105 months.

Few reports have described the imaging characteristics in detail. It typically consists of heterogeneous lesions, with variable signal intensities on T1- and T2-weighted images. This heterogeneity has most often been reported to represent the presence of blood degradation products. Depending on the age of the bleeding, variable T1- and T2-signals can be seen. T1-hyperintense spots or components have been reported in almost every available MRI report in literature, but were not found in this case [2,3,18,20,21], most probably due to the acute stage of the bleeding (1–2 days old). Perilesional edema was another frequent imaging feature [3,11,16,18,20,21]. Other reported imaging features include well-demarcated borders, hydrocephalus and ventriculomegaly, cystic areas within the lesion, and one case of intralesional calcifications [2,3,11]. Enhancement can be moderate or strong and has mainly been described as heterogeneous, explained by the presence of non-enhancing areas of hemorrhage in some reports [2,11,17,18,20,21]. It can be more pronounced in the periphery of the lesion. The enhancement patterns and other imaging features are summarized in Table 1. Conventional angiography has been performed in one case, in which spotty stains in the arterial phase were noticed with spreading and pooling of the contrast medium in the venous phase. The authors explained this by suggesting the presence of blood sinus-like structures in the mass [21].

**Table 1:** Reported imaging features in descending order (left) and reported enhancement patterns (right).

Imaging features	Enhancement patterns

Hemorrhage within lesion (any age)^2,3,16,18,20,21,c^	Intensive/strong^11,17,21^
Perilesional edema^3,11,16,18,20,21,c^	Moderate^19,c^
Well-demarcated lesion^2,11,21,c^	Heterogeneous^2,11,18,20,21,c^
(Focally) dilated ventricles^2,3,c^	Mainly periphery^2,20,c^
Cystic areas within lesion^3,c^	Periphery>>central^2,c^
Spontaneously hyperdense lesion^2,c^	Punctuated areas within the periphery^20^
Calcifications within lesion^2^	Moderate enhancement, mainly anteriorly^18^
	Intensive but heterogeneous^11,21^

c: current reported case.

The differential diagnosis is that of a hemorrhagic lesion, including cavernous malformation, glioblastoma, and metastasis [22,23,24,25,26].

The MRI features of a cavernous malformation typically consist of a lesion demonstrating a *T2-blooming sign*, due to a complete hypointense rim of hemosiderin, and a typical *popcorn ball* appearance on T2-weighted images, found in approximately 50–67% of the lesions [24]. Some lesions, however, have atypical features that can cause confusion, such as strong contrast enhancement, marked perilesional edema, mass effect, and signs of recent hemorrhage. A helpful feature to differentiate this lesion from other hemorrhagic lesions is the presence of a high T1-signal intensity (clearly hyperintense compared to normal white matter) within the perilesional vasogenic edema. This might be explained by the chronic leakage of red blood cells through the dysfunctional blood-brain barrier, causing chronic microbleeding. This theory also explains why the T1 hyperintensity is found adjacent to the lesion and not in the periphery of the vasogenic edema.

Glioblastoma multiforme (WHO grade IV astrocytoma) is the most common primary brain malignancy [25]. The prognosis of patients with this lesion is poor, with a median survival of less than 24 months. Glioblastoma is believed to have prothrombotic effects, rapidly causing areas of hypoxia and subsequent migration of tumor cells to the periphery of the lesion. This explains the typical manifestation of this lesion with rim enhancement and obvious central necrosis. This necrosis is much less present in primary angiosarcoma of the brain, in which small cystic areas have only been reported in two cases, including the current one. Secondary to necrosis, evidence of recent hemorrhage might be seen in glioblastoma, which might cause a diagnostic dilemma when the typical features are absent.

Tumors that frequently cause hemorrhagic intracranial metastases include choriocarcinoma, thyroid cancer, melanoma and renal cell carcinoma [26]. Cancer of the lung and breast can sometimes also cause hemorrhagic metastases. Because of their much higher prevalence, most hemorrhagic metastases originate from these two tumors. Approximately half of the brain metastases are solitary, possibly causing diagnostic difficulties. This is why scrutinizing the brain for smaller lesions is imperative. In most cases, a primary tumor is known, suggesting the diagnosis of a solitary brain metastasis. In general, patients with brain metastases are older than patients with a primary intracranial angiosarcoma. Their typical location at the gray-white matter junction might be an extra clue. Furthermore, they are often found at the border between vascular territories of the brain.

In conclusion, primary angiosarcoma of the central nervous system most commonly presents as a well-demarcated, heterogeneous, moderately to strongly enhancing lesion with signs of bleeding within the tumor and surrounding vasogenic edema.

## Competing Interests

The authors declare that they have no competing interests.
